# The Prophylactic Value of Gymnastics of the Jaw

**Published:** 1914-05-15

**Authors:** 

**Affiliations:** Elberfeld


### THE PROPHYLACTIC VALUE OF GYMNASTICS OF THE JAW.

BY DR. KLEINSORGEN, OF ELBERFELD.

The enormous prophylactic value of right use of the jaws in infancy is apt to be overlooked by the dental profession as a whole.

This neglect is no doubt due to the fact that prophylaxis can, in the nature of things, play but a very small part in lives spent in dealing with an overwhelming multitude of accidents. So far, its general principles and the business of disseminating these principles has been the concern of public health bodies. But that the accidents with which the dental profession is occupied are, nearly all of them, preventible, is a fact to which, within the profession, too great a prominence can scarcely be given.

While medical prophylaxis has to deal, even in the case of general orthopedics, with the most varied etiological moments, it is characteristic of dental prophylaxis to be obliged to deal with moments of *obstruction* or *false direction* in growth.

The percentage of inherited faulty development is small, and, for the purpose of our subject, negligible. The greater number of dental accidents originate mechanically and are due to our neglect of proper steering of the jaws in infancy.

In the first place, the use of the mouth after the first year as a sucking apparatus must be most strenuously opposed. In other words, milk, milky foods, broth and so on must be rigidly excluded. At the very best the utmost such foodstuffs can do is to excite the activity of the central part of the jaw. Such activity offers an opportunity for a snout-like development of the jaw.

Presuming the child to have been breast-fed, this moment, the moment of taking to solid foods is the first important mechanical moment. "Soft" fcods leave the sides of the jaw almost inactive. Development in breadth is materially retarded in favour of upward or forward growth. The immediate results—and their sequelae—of this faulty development upon the breathing apparatus and upon facial balance are only too well known. After the first half year, some solid food should be given. At the end of the first year, sucking should be absolutely excluded. *The child should chew itself tired.*

From the beginning of the second year the diet should consist principally of firm ripe fresh fruit and wholemeal bread-foods, either roast or baked. Drink, preferably milk, should be given after meals. Only a small quantity should be given, and it should be sipped slowly, or taken from a spoon.

It is of the greatest importance from the third year forward, from the time that nearly all the teeth have appeared, to see that the child *fills its mouth* at each bite. There is no other way of ensuring the complete activity of the sides of the jaw. The "small mouthful" occupies mainly the centre of the jaw, the well-filled mouth is fully occupied and must work hard.

The treatment of a child whose jaws have been neglected in infancy should include a systematic gymnastic for the jaw lasting for a quarter of an hour at least twice a day. During this practice, and of course at meal times, the use of the centre of the jaw should be discouraged in every possible way. A suitable chewing-gum may be used for the gymnastic, but a better medium is a hard carrot which may be chewed to pulp. The chewing of grain is also effective and wholesome.

The end in view is the working of the jaws twice a day to the point of fatigue. If this object is systematically attained from the sixth to the tenth year and, at the same time, soft foodstuffs are excluded from the diet, it is quite possible to prevent the development of anomalies which are already present owing to early neglect.

Considering the influence upon the young jaw of mechanical apparatus worn for months or years, as the case may be, it is not difficult to see that it can hardly fail to react to a rational gymnastic such as I have described.

In cases where with or without special attention to diet, good results have been obtained by mechanical treatment, the gymnastic is the surest means of making these results permanent. If it be neglected, and our bad habit of the "small mouthful" and of sucking soft foods in the centre of the jaw instead of chewing be allowed to prevail, there is every prospect of a return of the carefully corrected abnormalities. Later in life, say, from the beginning of the third decade, when the growth of the jaw is complete, these stern food rules may be relaxed to some extent, though not altogether, since an organ that is not sufficiently exercised tends towards deterioration. The flow of blood weakens, the process of nutrition is slower. Inactivity will show its results first in the weakest part. Not seldom this part is the thinnest part of the alveolar process—the results are familiar. For children, however, I strongly recommend the following ten rules of diet and mastication.

(1) Breast-feeding, if possible, for from six to eight months.

(2) With the appearance of the first tooth, and of instinctive chewing movements breast-feeding to be supplemented by half-solid foods.

(3) At the beginning of the second years all sucking movements to be excluded.

(4) "Dummies" and "comforts" of all kinds must be strictly avoided. The sale of these things should be forbidden by the public health authorities.

(5) From the beginning of the second year, half-fluid soups and broths, mealy foods and soft vegetables to be excluded.

(6) Hard foods should be given—bread, bread crusts, rusks and similar hard-bakes made from wholemeal flour, fruits in season, especially the various nuts.

(7) Instead of coral-rings and such things let the child have a good large hard carrot and chew until it is tired.

(8) When all the teeth are through, a *full* mouthful must be ensured in the interest of the complete activity of the sides of the jaw. It must be specially impressed upon the child that the front teeth are for biting and not for chewing.

(9) Drinking during meals must be absolutely forbidden. The saliva is, during chewing, a sufficiency of fluid. Half a glass of water or milk may be given, if desired, after a meal, which should end, if possible, with a juicy fruit. In most cases the fruit will be sufficient to allay thirst.

(10) Especially important is the prevention of chattering at meals. Chattering and drinking during meals are inimical to good digestion.—Abstracted from the *Deutsche Monatschrift fur Zahnheilkunde.*

